# A review of policy dissemination and implementation research funded by the National Institutes of Health, 2007–2014

**DOI:** 10.1186/s13012-015-0367-1

**Published:** 2016-01-04

**Authors:** Jonathan Purtle, Rachel Peters, Ross C. Brownson

**Affiliations:** 1 Department of Health Management & Policy, Drexel University School of Public Health, 3215 Market St., Philadelphia, PA 19104 USA; 2 Brown School, Division of Public Health Sciences, and Siteman Cancer Center, Washington University in St. Louis and Washington University School of Medicine, One Brookings Drive, Campus Box 1196, St. Louis, MO 63130 USA

## Abstract

**Background:**

Policy has a tremendous potential to improve population health when informed by research evidence. Such evidence, however, typically plays a suboptimal role in policymaking processes. The field of policy dissemination and implementation research (policy D&I) exists to address this challenge. The purpose of this study was to: (1) determine the extent to which policy D&I was funded by the National Institutes of Health (NIH), (2) identify trends in NIH-funded policy D&I, and (3) describe characteristics of NIH-funded policy D&I projects.

**Methods:**

The NIH Research Portfolio Online Reporting Tool was used to identify all projects funded through D&I-focused funding announcements. We screened for policy D&I projects by searching project title, abstract, and term fields for mentions of “policy,” “policies,” “law,” “legal,” “legislation,” “ordinance,” “statute,” “regulation,” “regulatory,” “code,” or “rule.” A project was classified as policy D&I if it explicitly proposed to conduct research about the content of a policy, the process through which it was developed, or outcomes it produced. A coding guide was iteratively developed, and all projects were independently coded by two researchers. ClinicalTrials.gov and PubMed were used to obtain additional project information and validate coding decisions. Descriptive statistics—stratified by funding mechanism, Institute, and project characteristics—were produced.

**Results:**

Between 2007 and 2014, 146 projects were funded through the D&I funding announcements, 12 (8.2 %) of which were policy D&I. Policy D&I funding totaled $16,177,250, equivalent to 10.5 % of all funding through the D&I funding announcements. The proportion of funding for policy D&I projects ranged from 14.6 % in 2007 to 8.0 % in 2012. Policy D&I projects were primarily focused on policy outcomes (66.7 %), implementation (41.7 %), state-level policies (41.7 %), and policies within the USA (83.3 %). Tobacco (33.3 %) and cancer (25.0 %) control were the primary topics of focus. Many projects combined survey (58.3 %) and interview (33.3 %) methods with analysis of archival data sources.

**Conclusions:**

NIH has made an initial investment in policy D&I research, but the level of support has varied between Institutes. Policy D&I researchers have utilized a variety of designs, methods, and data sources to investigate the development processes, content, and outcomes of public and private policies.

**Electronic supplementary material:**

The online version of this article (doi:10.1186/s13012-015-0367-1) contains supplementary material, which is available to authorized users.

## Background

Policy has a tremendous potential to improve population health [1, 2]. The extent to which policy produces such outcomes, however, often depends upon the degree to which it is informed by scientific knowledge and aligned with evidence-based interventions [3–5]. Unfortunately, research typically plays a minor role in policymaking processes [6, 7]. For decades, scholars have documented myriad barriers to translating research into policy—including political influences, economic constraints, scientific uncertainty, and institutional culture [8–20].

The field of health policy dissemination and implementation research (policy D&I) exists to address these challenges. Policy D&I is focused on generating knowledge to effectively spread research evidence among policymakers and integrate evidence-based interventions into policy designs [5]. Policy D&I, known as policy “knowledge translation and exchange” outside of the USA, has evolved over the past 40 years. The 1970s and 1980s were marked by interest in how social science research was used in public policymaking and saw the development of key contributions to the field—such as Weiss’ typology of research evidence [7] and Caplan’s two communities theory of knowledge utilization [17]. In the 1990s and 2000s, frameworks and instruments for policy D&I emerged [21–25] and the field was embraced within the discipline of public health [9, 26, 27]. Today, policy D&I is a priority of the World Health Organization [28, 29] and a major focus of journals such as *Health Research Policy and Systems* and *Evidence & Policy.*

The history of policy D&I has demonstrated that government funding is essential for a county to develop and maintain a robust policy D&I research infrastructure [22, 30]. Outside of the USA, governments have made sustained investments in policy D&I research and initiatives to promote the use of research evidence in health policymaking. Examples include the Canadian Institutes of Health Research’s integrated knowledge translation programs, the National Health and Medical Research Council of Australia’s Partnership Projects and Centres, and the UK National Institute for Health Research’s Collaboration for Leadership in Applied Health Research and Care. While national governments have invested in policy D&I abroad, policy D&I research has largely existed outside of the mainstream, government-funded health research enterprise in the USA [6]. This is potentially changing, however, as the National Institutes of Health (NIH)—the largest government funder of health research in the USA—has recently invested in the broader field of D&I research and identified policy D&I as a priority [31].

In 2002, the National Institute of Mental Health issued a Program Announcement (PAR) entitled “Dissemination and Implementation Research in Mental Health” that highlighted the importance of developing effective strategies to disseminate research findings to the policymakers [32]. In 2005, the PAR was re-issued under the broader name “Dissemination and Implementation Research in Health” (D&IRH) with more participating Institutes (e.g., the National Cancer Institute, National Institute on Drug Abuse) [33]. In 2007, the NIH Center for Scientific Review created a permanent D&IRH study section to evaluate D&I proposals, re-affirming NIH’s investment in the field [34]. In 2013, the “D&IRH” PAR was re-issued, this time with a greater emphasis on policy D&I research [35]. For example, the PAR encouraged applications that “address[ed] the complexity of bridging research, policy and practice.”

NIH has expressed interest in policy D&I research, but the extent to which it has funded research in this area is unclear. Two recent studies reviewed NIH D&I funding related to specific topics; one study focused on nursing [36] and the other focused on cancer control [37]. A similar review of policy D&I research is needed to assess growth of the field and identify funding gaps. Such a study would support growth in the field of policy D&I in the USA by providing investigators with a menu of approaches to policy D&I research that can be adopted and adapted when developing proposals. This article reports the results of a study that was conducted to address these knowledge gaps. The aims of the study were to: (1) determine the extent to which policy D&I research has been funded by NIH between 2007 and 2014, (2) identify trends in NIH-funded policy D&I research, and (3) describe the characteristics of NIH-funded policy D&I research projects.

## Methods

We used the NIH Research Portfolio Online Reporting Tool [38] to identify all projects that received funding during any US fiscal year between 2007 and 2014 through the D&I funding opportunity announcements (FOAs) Dissemination and Implementation Research in Health (PARs: 06-039, 06-071, 06-072, 06-520, 06-521, 07-086, 10-038, 10-039, 10-040, 13-054, 13-055, 13-056) or Dissemination and Implementation Research in Mental Health (PA 02-131). We selected 2007 as the starting point because it was the first year that projects supported by the Dissemination and Implementation Research in Health FOAs received funding. For each project, we extracted the title, abstract, project terms, award amount per fiscal year, funding mechanism, and Institute.

Our review was guided by Bogenschneider’s definition of policy as: “the development, enactment, and implementation of a plan or course of action carried out through law, rule, code, or other mechanism in the public or private sector” [5]. Accordingly, we identified potential policy D&I projects by searching the project title, abstract, and term fields for mentions of “policy,” “policies,” “law,” “legal,” “legislation,” “ordinance,” “statute,” “regulation,” “regulatory,” “code,” or “rule.” Two coders then independently reviewed project abstracts and developed preliminary coding categories to capture project characteristics. These categories reflected themes in that data, categories used in previous NIH D&I funding reviews [36, 37], and policy D&I scholarship [3–6, 8, 9]. The coders then jointly developed a coding guide and independently re-reviewed and coded the projects. Because many projects were not exclusively focused on policy D&I, projects were coded according to their policy D&I features. Incongruent coding decisions were identified in <10 % of projects and resolved through discussions.

We classified a project as policy D&I if it explicitly proposed to conduct empirical research about the “content” of a policy (e.g., analysis of the text of clean indoor air laws), the “process” through which it was developed (e.g., assessment of how state legislators use research evidence when developing clean indoor air laws), or the “outcomes” it produced (e.g., evaluation of the impacts of clean indoor air laws on cardiovascular health outcomes). These inclusion criteria were informed by Bogenschneider’s definition of policy [6] and domains of health policy research proposed by Brownson and colleagues [3–5]. For projects classified as policy D&I, we searched for its NIH project number in ClinicalTrials.gov [39] and PubMed [40] to obtain additional information and validate coding decisions.

We calculated the total dollar amount awarded through all NIH research grants and through the D&I FOAs, stratified by funding mechanism and Institute. We also calculated the amount awarded for policy D&I projects, and the percentage of total D&I FOA funding they comprised, within strata. Data were managed and analyzed in Microsoft Excel.

## Results and discussion

**Fig. 1:**
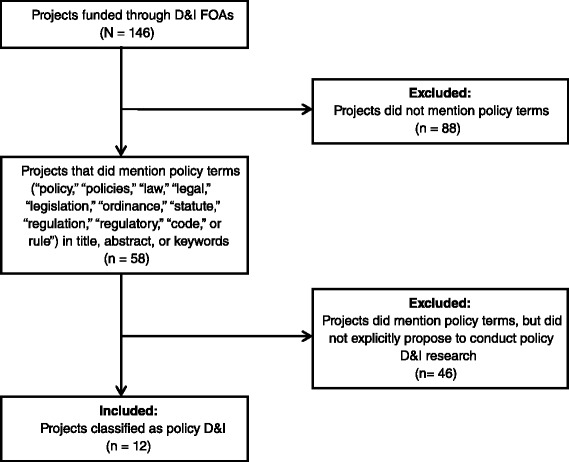
Flow diagram summarizing the process used to identify policy D&I research projects funded by NIH, federal fiscal years 2007–2014. *NIH* National Institutes of Health, *D&I* dissemination and implementation, *FOA*s funding opportunity announcements

Between 2007 and 2014, 146 projects were funded through the D&I FOAs, 12 (8.2 %) of which were classified as policy D&I research (Fig. 1). A total of $16,177,250 was awarded for these projects, equivalent to 10.5 % of all funding through the D&I FOAs (Table 1). The NIH Office of the Director allocated 69.7 % of its D&I FOA funding to a policy D&I project, indicating agency-level support for the field. The National Cancer Institute (NCI) was the primary funder of policy D&I research, supporting six projects which comprised 13.0 % of all NCI funding through the D&I FOAs. Some Institutes that were engaged in D&I funding—such as the National Institute of Nursing Research which funded seven D&I projects comprising over 1.0 % of the Institute’s total research grant spending, a proportion larger than any other Institute—did not fund any policy D&I projects. Differences in policy D&I funding between Institutes could potentially be a reflection of varying levels of knowledge about policy D&I research among investigators within different health science disciplines or differences in the degree to which the disciplines are clinically, as opposed to policy, oriented.

**Table 1:** NIH-funded policy D&I research projects by funding mechanism, Institute, and percentage of all research funded through D&I FOAs, federal fiscal years 2007–2014

Funding source	Number of projects funded through D&I FOAs	Amount funded through D&I FOAs (%)^a^	Number of policy D&I projects funded (%)^b^	Amount funded for policy of D&I (%)^b^
Total				
	146	$154,339,271 (0.09)	12 (8.2)	$16,177,250 (10.5)
Funding mechanism				
R01	82	$134,439,725 (0.19)	7 (8.5)	$14,626,930 (10.9)
R03	12	$1,745,967 (0.25)	1 (8.3)	$150,000 (8.6)
R21	51	$18,042,301 (0.36)	4 (7.8)	$1,400,320 (7.8)
R34	1	$111,278 (0.03)	0 (0)	$0 (0)
Institute				
NCI	46	$47,631,105 (0.19)	6 (13.0)	$5,993,592 (12.6)
NIMH	40	$51,237,349 (0.57)	3 (7.5)	$4,328,310 (8.4)
NIAID	18	$13,093,827 (0.06)	1 (5.6)	$320,720 (2.4)
NIDA	12	$11,691,400 (0.19)	1 (8.3)	$1,872,963 (16.0)
NHLBI	8	$12,248,387 (0.07)	1 (12.5)	$3,393,577 (27.7)
NINR	7	$8,983,908 (1.03)	0 (0)	$0 (0)
FIC	4	$667,849 (0.29)	0 (0)	$0 (0)
OD	3	$384,426 (0.02)	1 (33.3)	$268,088 (69.7)
NIDCR	3	$2,538,584 (0.11)	0 (0)	$0 (0)
NCCAM	2	$864,887 (0.17)	0 (0)	$0 (0)
NIDCD	2	$352,052 (0.01)	0 (0)	$0 (0)
NINDS	1	$2,601,603 (0.02)	0 (0)	$0 (0)
NIDDK	1	$1,396,497 (0.01)	0 (0)	$0 (0)
NIAAA	1	$408,039 (0.02)	0 (0)	$0 (0)
NIA	1	$239,358 (0.00)	0 (0)	$0 (0)

*NIH* National Institutes of Health, *NCI* National Cancer Institute, *NIMH* National Institute of Mental Health, *NIDA* National Institute on Drug Abuse, *NHLBI* National Heart, Lung, and Blood Institute, *OD* NIH Office of the Director, *NIAID* National Institute of Allergy and Infectious Diseases, *NINR* National Institute of Nursing Research, *FIC* Fogarty International Center, *NIDCR* National Institute of Dental and Craniofacial Research, *NCCAM* National Center for Complementary and Alternative Medicine, *NIDCD* National Institute on Deafness and Other Communication Disorders, *NINDS* National Institute of Neurological Disorders and Stroke, *NIDDK* National Institute of Diabetes and Digestive and Kidney Diseases, *NIAAA* National Institute on Alcohol Abuse and Alcoholism, *NIA* National Institute on Aging, *D&I* dissemination and implementation, *FOA*s funding opportunity announcements

^a^Percentages indicating the proportion of D&I funding within total NIH research grant spending category (e.g., proportion of research grant funding for R01 projects that were for D&I projects, $134,439,725/$71,808,892,375 = 0.19 %). Total research grant amounts for each NIH spending category are not shown. Source: https://report.nih.gov/fundingfacts/fundingfacts.aspx

^b^Percentages indicating the proportion of policy D&I within total D&I category (e.g., proportion of R01 projects funded through D&I FOAs that were policy D&I projects, 7/82 = 8.5 %)

**Fig. 2:**
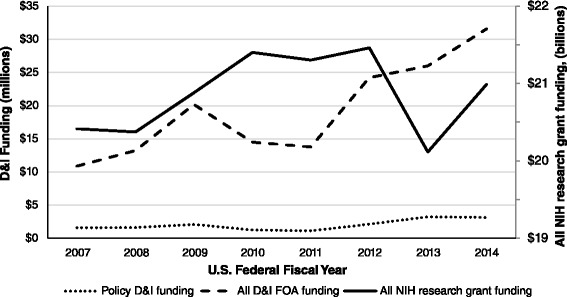
Annual trends in NIH funding for D&I research, federal fiscal years 2007–2014. “Policy D&I funding” and “all D&I FOA funding” in millions, “all NIH research grant funding” in billions, US dollars. *NIH* National Institutes of Health, *D&I* dissemination and implementation, *FOA*s funding opportunity announcements

NIH funding for policy D&I increased between 2007 and 2014 (Fig. 2). Annual policy D&I funding increased by 98.9 % within this period, from $1,584,327 in 2007 to $3,151,286 in 2014. The proportion of funding awarded through the D&I FOAs that was for policy D&I projects ranged from 14.6 % in 2007 to 8.0 % in 2011. Between 2012 and 2013, funding decreased by 6.3 % for all NIH research grants and increased by 7.4 % for projects funded through the D&I FOAs and by 51.8 % for policy D&I projects.

NIH-funded policy D&I projects utilized a range of methodologies and data sources across different domains of policy research (Tables 2, 3, Additional file 1). These projects were primarily focused on policy outcomes (66.7 %), implementation research (41.7 %), state-level policies (41.7 %), and the USA (83.3 %). Tobacco (33.3 %) and cancer control (25.0 %), for which evidence-based policy strategies exist [41, 42], were the most common topics of focus. Many projects combined survey (58.3 %) and interview (33.3 %) methods with analysis of archival data sources, such as medical records (25.0 %) and policy documents (25.0 %). It was unclear; however, the extent to which projects used true mixed-method designs (e.g., the results of one method informed data collection of the other, or the results of both methods were presented together).

**Table 2:** NIH-funded policy D&I research projects by study characteristics, federal fiscal years 2007–2014

Study characteristics	Number of policy D&I projects (%)^a^
	(*N* = 12)
Domain of policy research	
Outcome	8 (66.7 %)
Process	5 (41.7 %)
Content	1 (8.3 %)
Type of policy	
Public (e.g., laws, regulations)	6 (50.0 %)
Heath care financing/reimbursement	3 (25.0 %)
Workplace	2 (16.7 %)
Clinical practice	1 (8.3 %)
Level of policy	
State	5 (41.7 %)
Local	3 (25.0 %)
Organization	3 (25.0 %)
National	1 (8.3 %)
Region of research focus	
USA	10 (83.3 %)
Outside of USA	2 (16.7 %)
Health issue	
Tobacco	4 (33.3 %)
Cancer	3 (25.0 %)
Drug abuse	2 (16.7 %)
Mental health	2 (16.7 %)
Child welfare	2 (16.7 %)
Implementation science objectives	
Implementation	5 (41.7 %)
Dissemination	4 (33.3 %)
Adoption	2 (16.7 %)
Sustainability	1 (8.3 %)
Study design	
Quasi-experimental	5 (41.7 %)
Non-experimental	4 (33.3 %)
Experimental	3 (25.0 %)
Methods used	
Quantitative, surveys	7 (58.3 %)
Qualitative, interviews	4 (33.3 %)
Quantitative, medical record review	3 (25.0 %)
Content analysis, document analysis	3 (25.0 %)
Qualitative, focus groups	1 (8.3 %)
Quantitative, agent-based modeling	1 (8.3 %)
Content analysis, media analysis	1 (8.3 %)
Data source	
Policymakers	3 (25.0 %)
Policy documents	3 (25.0 %)
Healthcare providers	3 (25.0 %)
Administrative documents	3 (25.0 %)
Medical records	3 (25.0 %)
Community stakeholders	2 (16.7 %)
Media coverage	1 (8.3 %)
Unknown	1 (8.3 %)

*NIH* National Institutes of Health, *D&I* dissemination and implementation

^a^Some percentages exceed 100 because categories were not mutually exclusive

**Table 3:** NIH-funded policy D&I research projects by individual project details, federal fiscal years 2007–2014 (*N* = 12)

Project number	Title	Objective	Funding source	Start date	Country of focus
R01HL086450	An intervention for promoting smoke-free policy in rural Kentucky	To test the effects of a community intervention on smoke-free policy outcomes in rural underserved communities	NHLBI	April 1, 2007	US
R01CA124404	Cancer control dissemination research among state-level policy makers	To increase the dissemination of evidence-based interventions to control cancer, primarily focusing on the uptake of effective environmental and policy approaches among state-level policy makers	NCI	September 27, 2007	US
R01MH072961	Mixed methods study of EBP sustainment in a statewide service system	To examine factors that either support or limit sustainment of an evidence-based child neglect intervention in a large statewide public service system	NIMH	September 22, 2005	US
R01CA160327	Disseminating evidence-based interventions to control cancer	To increase the dissemination of EBPPs to control cancer, focusing on the uptake of effective approaches among state-level practitioners	NCI/OD	May 3, 2012	US
R01DA030431	To test a payer/treatment agency intervention to increase use of buprenorphine	To test whether clinician training and the use of organizational change strategies are sufficient for disseminating an evidence-based practice (EBP), or if changes to both organizational systems and payer policy result in greater EBP use	NIDA	March 1, 2012	US
R03CA128644	Translating science into policy: a survey of state tobacco control plans	To examine the structures and processes used by states to develop strategic plans to reduce tobacco use and prevent initiation	NCI	June 2, 2008	US
R21CA136435	Workplace health promotion	To enhance the dissemination potential of a successful intervention, Workplace Solutions that was developed to disseminate a set of 15 evidence - based cancer prevention strategies to workplaces	NCI	July 16, 2009	US
R01MH104200	Value-based purchasing in implementation of depression care in community clinics	To assess the role of value-based purchasing (VBP), a policy strategy, to enhance planned implementation of evidence-based care in CHCs	NIMH	August 1, 2014	US
R01CA175329	Implementing tobacco use treatment guidelines in community health centers in Vietnam	To fill the current research-to-practice gap by conducting a randomized controlled trial that compares the effectiveness and cost effectiveness of two practical and highly replicable strategies for implementing evidence-based guidelines for the treatment of tobacco use in public health clinics in Vietnam	NCI	September 30, 2013	Vietnam
R21MH098124	Development and validation of implementation climate measures	To develop measures of organizational climate, leadership, and provider behaviors likely to impact the implementation of evidence-based practices	NIMH	June 24, 2013	US
R21CA172938	A retail policy laboratory: modeling impact of retailer reduction on tobacco use	To examine the interplay between retailer density reductions and patterns of tobacco purchasing	NCI	July 1, 2013	US
R21AI095979	Sustainable financial incentives to improve prescription practices for malaria	To test an innovative, sustainable financial incentive designed to reduce the number of non-malarial fevers that are treated inappropriately with antimalarial drugs	NIAID	April 1, 2012	Kenya

*US* United States, *NIH* National Institutes of Health, *NCI* National Cancer Institute, *NIMH* National Institute of Mental Health, *NIDA* National Institute on Drug Abuse, *NHLBI* National Heart, Lung, and Blood Institute, *OD* NIH Office of the Director, *NIAID* National Institute of Allergy and Infectious Diseases, *D&I* dissemination and implementation

### Limitations

We limited our review to projects funded through FOAs explicitly focused on D&I and may not have identified all NIH-funded policy D&I projects. Our review also did not capture policy D&I projects funded by other US government agencies or philanthropies. We did not attempt to obtain the full text of proposals and thus did not have complete information on project characteristics, such as information on how the projects planned to disseminate their findings. We also did not have data on the characteristics of policy D&I research proposals that were unsuccessful in obtaining funding through NIH. Thus, we were unable to identify specific characteristics that increased the likelihood of a policy D&I project being funded. We dichotomously categorized projects as policy D&I (yes/no) and did not differentiate between projects in which policy D&I was the primary focus or subcomponent. Because only 12 policy D&I projects were identified, subgroup differences and comparisons should be interpreted with caution.

## Conclusions

NIH has made an initial investment in policy D&I research, signaling that the field might be entering the mainstream US health research enterprise. The level of support for policy D&I research, however, has varied between Institutes and is probably not commensurate with the potential of evidence-based policy to positively impact human health. Policy D&I researchers have utilized a variety of designs, methods, and data sources to investigate the development processes, content, and outcomes of public and private policies. By mapping the characteristics of NIH funded policy D&I research projects, the current study provides investigators with guidance on approaches to policy D&I research that they can consider when conceptualizing research ideas and developing NIH proposals in this area.

## Additional file

Additional file 1:
**NIH-funded policy D&I projects.** (XLSM 583 kb)
